# Automatic vertebrae localization and segmentation in CT with a two-stage Dense-U-Net

**DOI:** 10.1038/s41598-021-01296-1

**Published:** 2021-11-12

**Authors:** Pengfei Cheng, Yusheng Yang, Huiqiang Yu, Yongyi He

**Affiliations:** Shanghai Key Laboratory of Intelligent Manufacturing and Robotics, Shanghai University, Shanghai, 200444 China

**Keywords:** Biomedical engineering, Positron-emission tomography, X-ray tomography, Data processing, Image processing, Machine learning

## Abstract

Automatic vertebrae localization and segmentation in computed tomography (CT) are fundamental for spinal image analysis and spine surgery with computer-assisted surgery systems. But they remain challenging due to high variation in spinal anatomy among patients. In this paper, we proposed a deep-learning approach for automatic CT vertebrae localization and segmentation with a two-stage Dense-U-Net. The first stage used a 2D-Dense-U-Net to localize vertebrae by detecting the vertebrae centroids with dense labels and 2D slices. The second stage segmented the specific vertebra within a region-of-interest identified based on the centroid using 3D-Dense-U-Net. Finally, each segmented vertebra was merged into a complete spine and resampled to original resolution. We evaluated our method on the dataset from the CSI 2014 Workshop with 6 metrics: location error (1.69 ± 0.78 mm), detection rate (100%) for vertebrae localization; the dice coefficient (0.953 ± 0.014), intersection over union (0.911 ± 0.025), Hausdorff distance (4.013 ± 2.128 mm), pixel accuracy (0.998 ± 0.001) for vertebrae segmentation. The experimental results demonstrated the efficiency of the proposed method. Furthermore, evaluation on the dataset from the xVertSeg challenge with location error (4.12 ± 2.31), detection rate (100%), dice coefficient (0.877 ± 0.035) shows the generalizability of our method. In summary, our solution localized the vertebrae successfully by detecting the centroids of vertebrae and implemented instance segmentation of vertebrae in the whole spine.

## Introduction

The vertebra, which is one of the main components of the spine, plays an important role in supporting the human body’s walk, twist and move. The structure of the vertebra is very complicated, and its state has an essential influence on the body’s health. Identifying the pathologies of the vertebra not only helps to prevent the deterioration of the spine-related disease in the early phase of the treatment but also provides essential information for the doctor to design the therapeutic schedule. One common approach to acquire the status of the vertebra is scanning it with the computed tomography (CT) technology, and the captured CT spinal images are used in the subsequent pathology analysis. However, the shape of the vertebra is irregular, and its architecture varies among different people. Furthermore, the adjacent vertebrae and ribs have similar structures. All these factors post challenges for localizing the vertebra and segmenting the vertebra from CT images.

Vertebrae localization and segmentation from CT spinal images are fundamental for spine image analysis and 3D spine reconstruction applications, such as identifying spine abnormalities^1^, photogrammetry-based biomechanical modeling^2^, and image-guided spine intervention^3^. Since there are many slices, i.e., images, for CT scanning, localizing and segmenting vertebrae manually will be very time-consuming, and the inter- and intra- observer errors are inevitable among different operators. In the past decades, many automatic localization and segmentation methods were proposed to improve localization precision and increase the segmentation efficiency.

For vertebrae localization, traditional methods usually combine random forests with other statistical graphical models^4,5^ and appearance information^6^. Due to the advances of deep learning^7^, recent best-performing methods for vertebrae localization are based on convolutional neural networks (CNNs). In 2017, Yang et al^8^ generated predictions for vertebrae localization by incorporating a pre-trained model of neighboring landmarks into their CNN. Liao et al.^9^ published a solution that regresses the centroids of the vertebrae using a CNN and recurrent neural network (RNN) to capture the order of the vertebrae and to incorporate long-range contextual information. One of the state-of-the-art methods was proposed by McCouat et al.^10^ who improves the accuracy of the vertebrae centroid detection and localization with a revised approach to dense labeling from sparse centroid annotations.

For vertebrae segmentation, the early approaches typically are based on traditional image processing methods that could be classified into region growing methods^11^, the level set method^12^, clustering approaches^13^, energy minimization methods^14^, statistical shape model methods^15^, atlas-based methods^16^, etc. After some CT spine datasets were public^17^, researchers began to combine deep-learning methods with statistical modeling or other traditional methods which showed better performance^18,19^. Recently published vertebrae segmentation methods have replaced explicit modeling of the vertebral shape and appearance with convolutional neural networks. For example, Zhou et al.^20^ described an N-shaped 3D fully convolution network (FCN). Kolařík et al^21^ validated the superior performance of 3D-Dense-U-Net in medical image segmentation. But both Zhou et al.^20^ and Kolařík^21^ failed to separately segment vertebrae from the adjacent vertebrae in their work.

There are also some researchers implementing sequentially localization and segmentation with two stage method in their work. Sekuboyina et al.^22^ proposed a two-staged approach that, the first stage located the lumbar region using the global context and the second stage, exploited the local context in the localized lumbar region to segment and label the lumbar vertebrae. However, solely projected 2D views of the 3D spinal anatomy were used as the input of their networks. It reduces the amount of information that needs to be processed, but beneficial volumetric information may be lost. Janssens et al.^23^ relied on two consecutive networks, first using a regression CNN to estimate a bounding box of the lumbar region, followed by a classification CNN to perform voxel labeling within that bounding box to segment the lumbar vertebrae. Lessmann et al.^24^ presented an iterative CNN for successively localizing and segmenting vertebra instance-by-instance, while the network needs to incorporate information of already segmented vertebrae.

**Figure 1:**
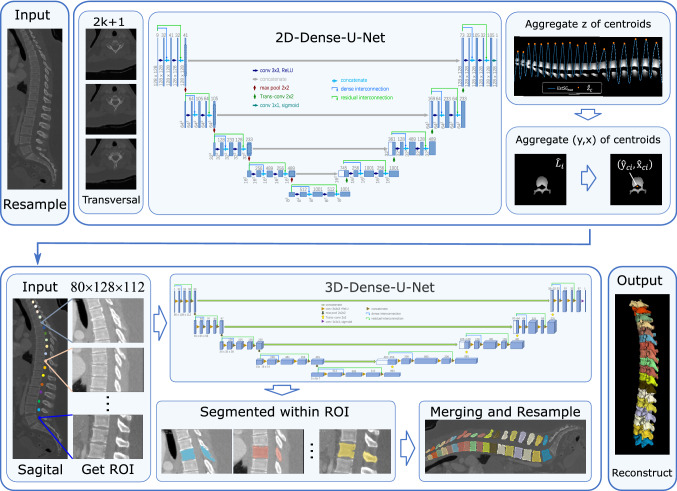
Overview of our proposed automatic vertebrae localization and segmentation method with a two-stage Dense-U-Net.

In this paper, we implemented a complete process to automatically localize and segment vertebrae by proposing a two-stage Dense-U-Net as illustrated in Fig. 1. At the first stage, by creating sparse annotation of vertebrae centroids and converting them to dense labels, we built a dataset from the original dataset for vertebrae localization. Then, combing an aggregating method to postprocess the predicted result, the centroid of the vertebrae in each CT image can be predicted with a 2D-Dense-U-Net, and this information is treated as the prior for the subsequent instance segmentation. At the second stage, a 3D-Dense-U-Net segmented the specific vertebrae within the region-of-interests (ROIs) that are identified with the prior centroid information. Merging the individual segmented vertebrae in physiology sequence, the whole shape of the spine can be captured accordingly. We tested the proposed method on two datasets from CSI 2014 Workshop^25^ and xVertSeg challenge^26^ on the SpineWeb^17^, the former experimental results showed the efficiency of our solution and the later showed the generalizability.

## Material and methods

In this section, we first introduce the datasets used in this paper. Then, methods used for vertebrae localization and vertebrae segmentation are presented respectively in detail incl. data preparation for training and testing, Dense-U-Net architecture, and postprocessing of predicted results.

### Dataset

**Figure 2:**
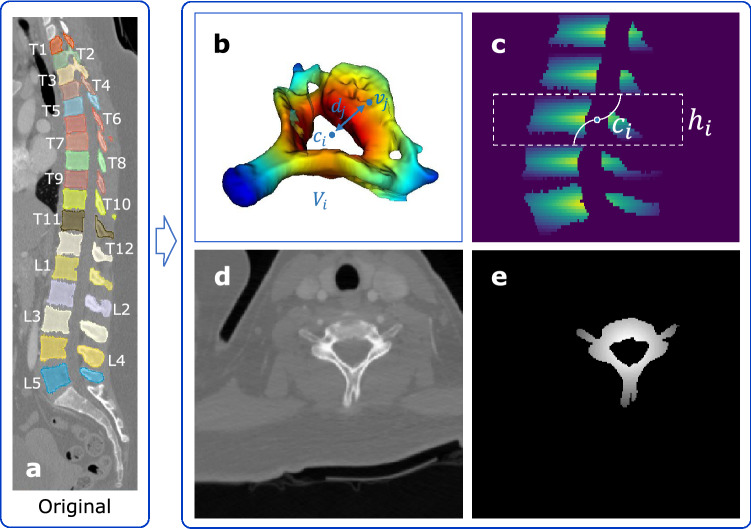
(**a**) The position of each vertebra and its corresponding label of CSI dataset. (**b**) A vertebra with its centroid. (**c**) A partial sagittal dense label of vertebrae centroids. (**d**), (**e**) Transversal slice and its corresponding dense label respectively used as the input and output of 2D-Dense-U-Net.

The CT spinal datasets used in this work are provided in mhd/raw format from CSI 2014 Workshop^25^ and xVertSeg challenge^26^ on the SpineWeb^17^, which is a collaborative platform of spine images. We used the dataset from CSI 2014 Workshop (CSI dataset) to evaluate the efficiency of our method and the dataset from xVertSeg challenge (xVertSeg dataset) to evaluate the generalizability of our method. The CSI dataset consists of 15 healthy cases that contain all thoracic and lumbar vertebrae and we divided them into two parts: case 1-10 for training and case 11-15 for testing. The position of each vertebra and its corresponding label are shown in Fig. 2a. The xVertSeg dataset contains 15 lumbar spine CT images incl. non-fractured and fractured vertebrae of which corresponding vertebra segmentation labels and fractured grade are also provided. Therefore, it could be also used to evaluate the performance on pathological cases. We divided them into two parts: 10 images for training and 5 for testing. The in-plane resolution and the slice thickness of the datasets are different. To reduce the inconsistency between different images and facilitate the convolution operation to extract common features, all spine CT images were resampled to an isotropic resolution of $1 \times 1 \times 1 \mathrm {~mm}^{3}$ per voxel using linear interpolation for the image and nearest interpolation for the label.

**Table 1:** Algorithm 1: Pseudocode of building dataset for vertebrae localization.

**Input:** *V*, Ground-truth labels of vertebrae in 3D spine CT images	
**Output:** *c*, The coordinates of vertebrae centroids *L*, ground-truth dense labels of vertebrae centroids in 3D spine CT images	
01: **for** each $i \in \left[ 0, n_{v}\right]$ **do**	
02: $c_{i} \leftarrow$ Get Centroid of $V_{i}$ in 3D Slicer^27^	
03: $c.{\text {append}} \left( c_{i}\right)$	
04: $d_{\max } \leftarrow$ Get the approximated radii of each vertebra $V_{i}$^28^	
05: **for** $v_{j}$ in $V_{i}$ **do**	
06: $\quad d_{j} \leftarrow \left\| v_{j}-c_{i}\right\|$	
07: **if** $d_{j}>d_{\max }$ **then** $d_{j} \leftarrow d_{\max }$	
08: $\quad p_{j} \leftarrow$ Get $p_{j}$ by Eq. 1	
09: $\quad v_{j} \leftarrow \frac{p_{j}}{d_{\max }} \times 255$	
10: $\quad L_{i} .{\text {append}} \left( v_{j}\right)$	
11: **end for**	
12: $L.{\text {append}} \left( L_{i}\right)$	
13: **end for**	

### Vertebrae localization

#### Data preparation

At the first stage, we localized the vertebrae through a 2D-Dense-U-Net to detect the centroid of each vertebra. Since both datasets used in this paper only contain the labels of the vertebrae and come without vertebrae centroids (sparse labels), we built dense datasets from the original ones by creating sparse annotation of vertebrae centroids and converting them to dense labels. The building algorithm is inspired by McCouat et al.^10^ and shown in detail as in Table 1 Algorithm 1. Especially, to distinguish the vertebrae from each other in transversal direction obviously, a coefficient $p_{j}$ given by

1 $$\begin{aligned} p_{j}=\left( d_{\max }-d_{j}\right) \times \left( 1-\tan \left| \frac{z \text{ of } v_{j}-z \text{ of } c_{i}}{h_{i}}\right| \times \frac{\pi }{2}\right) \end{aligned}$$

was taken to keep the center slice of the vertebra more focused than other adjacent slices, where $d_{\max }$ is the approximated radii of the *i*th vertebra $V_i$, $v_j$ is the coordinate of the *j*th pixel in the *i*th vertebra $V_i$, $c_i$ is the coordinate of vertebra centroid of the ith vertebra $V_i$, *z* is the z component of the coordinate, $d_j$ is the Euclidean distance between $v_j$ and $c_i$, $h_i$ is the approximated height of the *i*th vertebra $V_i$. The symbols that appear in Algorithm 1 and Fig. 2 have the same meaning as aforementioned. A vertebra with its centroid is shown in Fig. 2b. A partial sagittal dense label of vertebrae centroids is shown in Fig. 2c. Transversal slice and its corresponding dense label respectively used as the input and output of 2D-Dense-U-Net are shown in Fig. 2d and e.

**Figure 3:**
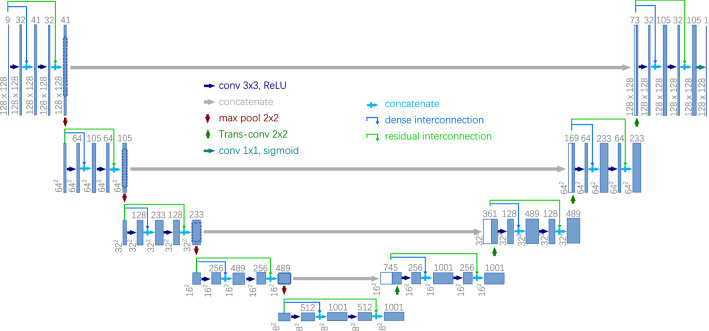
2D-Dense-U-Net architecture for vertebrae localization.

**Figure 4:**
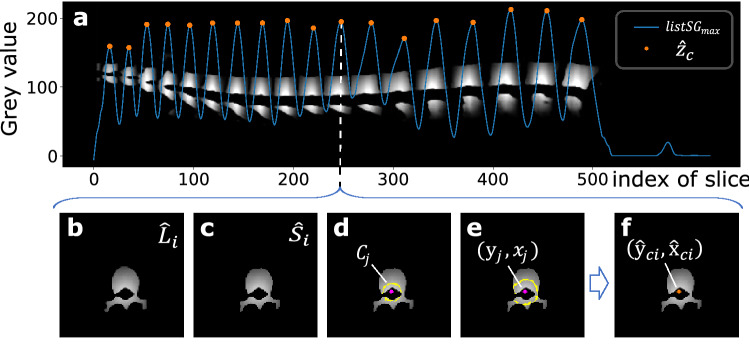
Aggregating method to estimate vertebra centroid.

#### 2D-Dense-U-Net architecture

The 2D-Dense-U-Net architecture for vertebrae localization is presented in Fig. 3. It is designed by adding interconnections to the U-Net architecture^29^, incl. residual interconnections (green links in Fig. 3) to transmit information over whole down or up-sampling blocks and dense interconnections (blue links in Fig. 3) to pass unprocessed information to the middle layer of down and up-sampling blocks. This is advantageous to improve the accuracy since these structures not only efficiently alleviate the vanishing gradient problem and strengthen feature propagation but also transfer back the fine-grained detail that otherwise would be lost in the down-sampling path. To cover 3D information in the 2D network, the input of the network is designed as 2k + 1 slices (k represents the amounts of slices, it is set as 4 in this paper) generated from one transversal slice (as shown in Fig. 2d) and its 2k adjacent slices. In particular, if the slice is at the start or the end of the spine CT images in the transversal direction, the missing adjacent slices are filled with zero. Each slice (In Fig. 2d) has a dense label (In Fig. 2e), containing 0s (for background) and floating-point numbers between 0 and 1 (for different proximity between each pixel and the centroid of the vertebra), for the network to learn from. At last, sigmoid activation was used on the output network layer and the binary cross-entropy was used as a loss function for the network.

**Table 2:** Algorithm 2: Pseudocode for aggregating the dense results to estimate each vertebra centroid.

**Input:** $\widehat{L}$, Predicted dense results of vertebrae centroids in 3D spine CT images	
**Output:** $\hat{c}\left( \hat{z}_{c}, \hat{y}_{c}, \hat{x}_{c}\right)$, Coordinates of aggregated vertebrae centroids	
01: **for** each $i \in \left[ 0, n_{s}\right]$ **do**	
02: $\quad v_{\max i} \leftarrow$ Get maximum of each slice $\widehat{L}_{i}$	
03: $\quad list_{\max } .{\text {append}}\left( v_{\max i}\right)$	
04: **end for**	
05: $listSG_{\max } \leftarrow$ Apply Savitzky-Golay filter^30^ to $list_{\max }$	
06: $\hat{z}_{c} \leftarrow$ Get peaks of $listSG_{\max }$	
07: **for** each $i \in \hat{z}_{c}$ **do**	
08: $\quad \hat{S}_{i} \leftarrow$ Apply a threshold to $\widehat{L}_{i}$	
09: $\quad S_{\max }, S_{\min } \leftarrow$ Get maximum and minimum of $\hat{S}_{i}$	
10: **for** each $j \in [2,6)$ **do**	
11: $\quad \quad C_{j} \leftarrow$ Get values in the range $\left( S_{\min } \times 0.1 \times j+S_{\max } \times 0.1 \times (10-j)\right) \pm 5$ of $\hat{S}_{i}$	
12: $\quad \quad \left( y_{j}, x_{j}\right) \leftarrow$ Fit the center of $C_{j}$ with least-squares method	
13: $\quad \quad y. {\text {append}} \left( y_{j}\right) , x . {\text {append}}\left( x_{j}\right)$	
14: **end for**	
15: $\quad \left( \hat{y}_{c i}, \hat{x}_{c i}\right) \leftarrow$ Get the mean value of (*y*, *x*)	
16: $\quad \hat{y}_{c} . {\text {append}} \left( \hat{y}_{c i}\right) , \hat{x}_{c} . {\text {append}}\left( \hat{x}_{c i}\right)$	
17: **end for**	
18: $\hat{c} \leftarrow \left( \hat{z}_{c}, \hat{y}_{c}, \hat{x}_{c}\right)$	

#### Postprocessing

After the dense results (As in Fig. 2e) are deduced from 2D-Dense-U-Net, these results are aggregated to estimate the vertebrae centroids by Algorithm 2 as shown in Table 2. As depicted in Fig. 4, first, the max gray value $v_{\max i}$ of each slice is calculated to make a complete curve $list_{\max }$. Second, the Savitzky-Golay filter^30^ is applied to filter out outliers and obtain a smoothed curve $listS G_{\max }$. Third, peaks of the curve $listS G_{\max }$ are captured as the coordinates $\hat{z}_{c}$ of the predicted centroids which represent the position of the nearest transversal slices to their centroids as depicted in Fig. 4a. Forth, to filter out some smaller erroneous predictions produced by the network, we apply a threshold of 50 on each slice $\hat{L}_{i}$ (in Fig. 4b) on coordinates $\hat{z}_{c}$ and obtain the thresholded slice $\hat{S}_{i}$ (in Fig. 4c). Then, we extract five circle-like contours $C_{j}$ (in Fig. 4d) between the maximum $S_{\max }$ and minimum $S_{\min }$ of the slice $\hat{S}_{i}$ and fit the centers $\left( y_{j}, x_{j}\right)$ (in Fig. 4e) of these contours by the least-squares method. Finally, the mean coordinates $\left( \hat{y}_{c i}, \hat{x}_{c i}\right)$ (in Fig. 4f) of these centers are taken as the coordinates y and x of the final predicted vertebra centroid, respectively.

### Vertebrae segmentation

#### Data preparation

**Figure 5:**
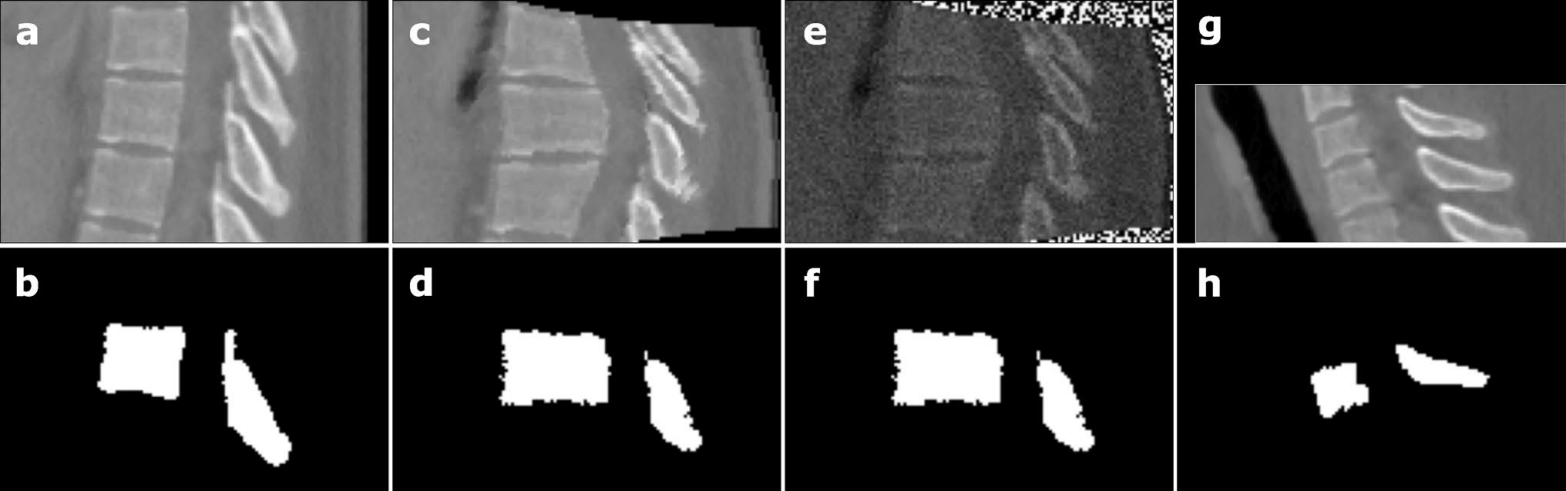
(**a**), (**b**) Sagittal image and label of ROI for segmentation. (**c**), (**d**) Elastically deforming of ROI. (**e**), (**f**) Adding Gaussian noise to ROI, (**g**), (**h**) Filling with 0s (black) beyond the boundary.

To further segment each vertebra, the ROI of each vertebra needs to be identified at the second stage. Based on the final centroid estimates from the first stage, we cropped ROIs from the resampled dataset with size $z \times y \times x(80 \times 128 \times 112)$ for images and its ground-truth labels, respectively as shown in Fig. 5a, b. To avoid overfitting and increase the amount of 3D spine CT images, data augmentation techniques are adopted. First, we elastically deform each ROI using the elastic deform python package^31^ on a $3 \times 3 \times 3$ grid as shown in Fig. 5c, d; Second, after elastically deformed, Gaussian noise with $\mu$ as the mean and $\sigma$ as the standard deviation was added to the ROIs, where $\mu = 0$ and variance $\sigma$ obeys the uniform distribution U (0, 0.1) as shown in Fig. 5e, f. Especially, if the ROI covers the region beyond the boundary of the 3D spine CT images, the outside part was filled with 0s (black) as shown in Fig. 5g, h.

**Figure 6:**
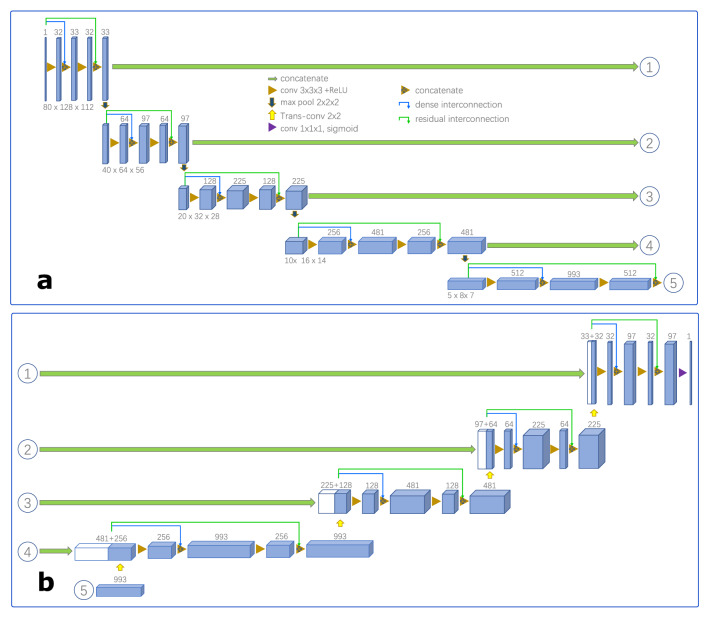
3D-Dense-U-Net architecture for vertebrae segmentation (Blue boxes represent feature maps. The number of channels is denoted above each feature map. The numbers in the circle from 1 to 5 are joints between (**a**) and (**b**)). (**a**), (**b**) The contracting and expansive path of 3D-Dense-U-Net.

#### 3D-Dense-U-Net architecture

To segment the vertebrae within the ROIs, 3D-Dense-U-Net is designed based on original U-Net implementation^29^ and 3D U-Net version^32^ but with added interconnections between layers processing the same feature size as shown in Fig. 6. To maintain the resolution of the figure, the contracting and the expansive path of 3D-Dense-U-Net are separately depicted in Fig. 6a and b, and the numbers in the circle from 1 to 5 are joints between them. The interconnections also include residual interconnection (green links in Fig. 6) to transmit information over whole down or up-sampling blocks and dense interconnections (blue links in Fig. 6) to pass unprocessed information to the middle layer of down and up-sampling blocks but in 3D mode. We used sigmoid activation on the output network layer and binary cross-entropy as the loss function, the output of the network is not labeled by just discrete values i.e., 0 or 1, but with continuous values in the range from 0 to 1. Therefore, after prediction, we used thresholding as post-processing on predicted data. Considering the different size of vertebrae and small size vertebra may lose information in respective large ROI , all pixels lesser than 0.5 were labeled as 0 and greater than 0.5 as 1 for T1 to T9; all pixels lesser than 0.9 were labeled as 0 and greater than 0.9 as 1 for T10 to L5. Because the ROI contains adjacent vertebrae which may cause some artifacts in the prediction, we had to threshold the predicted result to remove all stand-alone objects smaller than 500 voxels. This ensured the quality output without any artifacts in the segmented image. Through these steps, vertebrae are successfully segmented from the background and the adjacent vertebrae by 3D-Dense-U-Net within the ROIs.

#### Postprocessing

Finally, the predicted vertebrae were merged into a complete spine and resampled to original resolution. Moreover, to better display the segmented result and interaction with surgeons, the whole spine was reconstructed in 3D. Especially, since the segmentation of adjacent vertebrae is separated and independent, one pixel may be assigned to both vertebrae. To solve this conflict, in merging process, we created an empty CT scan, then each segmented vertebra is sequentially assigned to the empty CT scan based on the coordinates of its vertebral centroids pixel by piexl with the condition that the position of corresponding pixel is empty. In summary, with the mode of first localization in 2D slices and then segmentation in 3D ROIs, we finished vertebrae instance segmentation and didn’t need to process the whole spine CT images in the segmentation task, so the usage of GPU and memory could be saved and spatial semantic information for vertebrae segmentation isn’t lost.

## Experiments and results

### Experiment setup

Our experiments were conducted on a workstation operated under Ubuntu 20.04 system. The workstation is embedded with an Intel(R) Xeon(R) CPU, 64 GB memory, and two NVIDIA GeForce GTX 1080Ti GPU using CUDA 11.0. Our network was implemented in Keras 2.4.3 with TensorFlow 2.4.0 as the backend in the Python 3.8 environment. Specifically, as for the parameters in the training, we set the batch size as 1 and adopted the Adam optimizer^33^ with the learning rate equals to $10^{-5}$, beta1 to 0.9, beta2 to 0.999, epsilon to $10^{-8}$ and decay to $1.99 \times 10^{-7}$ separately. The epochs processed by the 2D-Dense-U-Net and 3D-Dense-U-Net are 30 and 50, respectively.

### Evaluation criteria

The result of vertebrae localization was evaluated in terms of the location error (LE) and detection rate (DR). Specifically, LE represents the Euclidean distance between the predicted centroid $\hat{c}$ and ground-truth centroid *c* of the vertebra and the DR means the proportion of the vertebra contained in the ROI and its whole vertebraas repectively as given in

2 $$\begin{aligned} L E(c, \hat{c})=\Vert c-\hat{c}\Vert ,\quad D R\left( V_{R O I}, V\right) =\frac{V_{R O I}}{V} \times 100 \%, \end{aligned}$$

where $\Vert c-\hat{c}\Vert$ means the Euclidean distance between *c* and $\hat{c}$, $V_{R O I}$ represents the partial vertebra contained in the ROI, *V* represents the whole vertebra.

As for the accuracy of vertebrae segmentation, four different criteria, incl. the dice coefficient (DC)^34^, the intersection over union (IoU)^35^, the Hausdorff distance (HD)^36^, and the pixel accuracy (PA)^37^ were evaluated. All results were computed by using the Visceral segmentation tool^38^. The DC and IoU that represent the amount of spatial overlap between the predicted region and the ground-truth region are calculated in different ways as

3 $$\begin{aligned} DC(X, Y)=\frac{2|X \cap Y|}{|X|+|Y|},\quad {\text {IoU}}(X, Y)=\frac{|X \cap Y|}{|X \cup Y|}, \end{aligned}$$

where *X* and *Y* stand for the number of positive pixels/voxels on the ground-truth and predicted result, separately.

The HD, which describes the distance between each surface voxel of the segmented surface *P* from the closest surface voxel in the ground-truth *G*, is defined by

4 $$\begin{aligned} H D(G, P)=\max (h(G, P), h(P, G)), \quad h(G, P)=\max _{g \in G} \min _{p \in P}\Vert g-p\Vert , \end{aligned}$$

where *h*(*G*, *P*) is called the directed Hausdorff distance, $\Vert g-p\Vert$ means the Euclidean distance between g and p.

The last criterion for vertebra segmentation is the PA, as given in

5 $$\begin{aligned} P A=\frac{T P+T N}{T P+F P+T N+F N}, \end{aligned}$$

where TP stands for true positive pixels or voxels, TN means true negative, FP means false positive, and FN represents the false negative.

### Results and discussion

Since the proposed approach was carried out in two-stage, their experiments were conducted and evaluated separately on CSI dataset. First, we evaluated the accuracy of vertebrae localization; next, the second experiments respectively evaluating the accuracy of vertebrae segmentation qualitatively and quantitatively were conducted and the results were also compared with some state-of-the-art methods. Moreover, to further evaluate the generalizability and the performance on pathological cases, we conducted experiments on xVertSeg dataset in terms of evaluation on LE, DR and DC.

**Figure 7:**
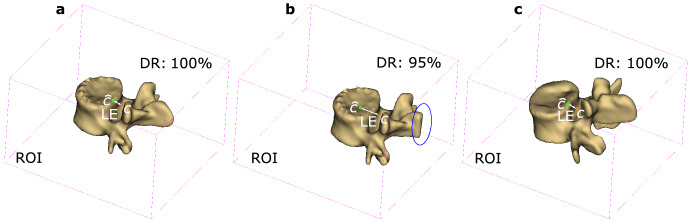
Visual demonstration of different LE and DR (the purple box is the identified ROI according to the predicted vertebra centroid $\hat{c}$). (**a**) DR: 100%. (**b**) DR: 95%. (**c**) ROI identified by the predicted vertebra centroid of case15/L3.

For vertebrae localization, the predicted vertebra centroid $\hat{c}$ at the first stage is used for identifying the ROI for subsequent vertebrae segmentation. If the location of vertebra centroid $\hat{c}$ is wrongly predicted, the ROI may only contain partial vertebra and result in information being lost. Thus, the location errors and detection rates were adopted to evaluate whether the ROI contained the whole vertebra as shown in Fig. 7. Figure 7a shows that the whole vertebra is contained in the ROI if the LE is small i.e., DR is 100% and by contrast Fig. 7b shows that too large LE (DR is 95%) causes the ROI only contains partial vertebra, and some valid information lost as shown in the blue oval circle. The location errors of all predicted vertebrae centroids are presented in Table 3. The mean location error of each vertebra is concluded in the last column “All” and all of them are under 3 mm. The mean location error of each case is concluded in the last row “Mean”. It can be found that the mean location error among five testing cases is 1.69 ± 0.78 mm. The maximum location error appears in case 15/L3 which is 4.35 mm, therefore, the ROI identified by its predicted centroid is visually demonstrated as in Fig. 7c. Although the location error of case15/L3 is the largest, the DR is still 100%, which means that the ROI still contains the whole vertebra. Furthermore, the detection rates of five testing cases were evaluated as shown in Table 4. It indicates that the detection rates are 100% for all cases, i.e., there is no valid information loss and all ROIs can be used as the input for the subsequent vertebrae segmentation.

**Table 3:** The location errors of the predicted vertebrae centroids.

LE	Case 11 (mm)	Case 12 (mm)	Case 13 (mm)	Case 14 (mm)	Case 15 (mm)	All (mm)
T1	1.76	1.65	2.15	0.69	1.94	1.64
T2	0.96	2.22	2.12	1.25	2.30	1.77
T3	1.45	0.88	1.40	2.31	1.54	1.52
T4	1.19	2.52	0.72	0.55	1.81	1.36
T5	2.54	2.51	1.40	2.26	0.95	1.93
T6	**0.47**	2.00	2.11	1.30	1.38	1.45
T7	1.35	**0.69**	1.42	1.41	**0.83**	1.14
T8	**2.66**	1.17	1.10	**0.40**	0.97	1.26
T9	1.36	2.45	**0.65**	0.22	0.84	**1.10**
T10	1.16	2.07	2.66	0.85	2.30	1.81
T11	1.42	2.78	2.86	1.12	2.94	2.22
T12	1.04	**3.31**	2.72	2.06	1.81	2.19
L1	2.38	1.40	0.94	1.31	1.98	1.60
L2	0.97	1.89	1.99	1.15	1.8	1.56
L3	1.64	1.79	**2.90**	2.01	**4.35**	**2.54**
L4	1.69	1.04	2.78	0.85	2.87	1.85
L5	0.74	1.56	2.30	**2.75**	1.98	1.87
Mean	1.46 ± 0.61	1.88 ± 0.72	1.90 ± 0.77	1.32 ± 0.73	1.92 ± 0.90	1.69 ± 0.78

Bold values indicates maximum and minimum values of the corresponding column or row.

**Table 4:** The detection rates of vertebrae localization.

	Case 11	Case 12	Case 13	Case 14	Case 15
DR	100%	100%	100%	100%	100%

To demonstrate the effectiveness and accuracy of the proposed vertebrae localization method, we also compared the location error of thoracic and lumbar with several start-of-the-art methods, incl. Chen et al.^39^, Liao et al.^9^, and McCouat et al.^10^. As presented in Table 5, the location errors of our method are smaller than other methods both in thoracic, lumbar and mean value of all vertebrae (row “Mean”). However, the dataset we used is different from the dataset used by the compared methods, since all of them conducted their methods on the dataset that is only for vertebrae localization and identification^5^ that can’t be used for our subsequent segmentation task. Therefore, the result only represents that we localized the centroids effectively and reached the accuracy of the state-of-the-art on our refined dataset. In summary, the first stage 2D-Dense-U-Net can localize the vertebrae successfully by detecting the vertebrae centroids and the accuracy of localization can provide valid ROIs for subsequent segmentation.

**Table 5:** Comparison of location errors on thoracic and lumbar.

	Chen et al.^39^	Liao et al.^9^	McCouat et al.^10^	Our Method
Thoracic	11.39 ± 16.48	7.78 ± 10.17	6.61 ± 7.40	1.62 ± 0.75
Lumbar	8.42 ± 8.62	5.61 ± 7.68	5.39 ± 8.70	1.88 ± 0.82
Mean	8.82 ± 13.04	6.47 ± 8.56	5.60 ± 7.10	1.69 ± 0.78

**Figure 8:**
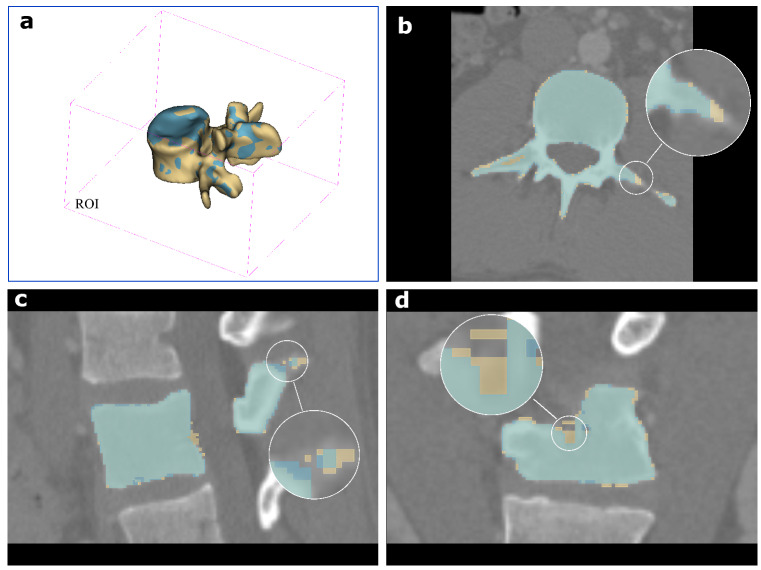
Visual demonstration of the predicted (blue) and the ground-truth (yellow) of case15/L3. (**a**) 3D model. (**b**) Transversal plane. (**c**) Sagittal plane. (**d**) Coronal plane.

For vertebrae segmentation, each ROI of the vertebra was identified according to the predicted vertebra centroid $\hat{c}$. Then, the ROI was fed into the 3D-Dense-U-Net for vertebra segmentation. Taking case15/L3 as a visual example, the predicted result and the corresponding ground-truth are demonstrated in Fig. 8. It shows that 3D-Dense-U-Net successfully segmented the vertebra from the background and the adjacent vertebrae within the ROI. However, the result also shows that there are still some pixels that were not correctly predicted (pixels nonoverlapping in 3D model, transversal plane, sagittal plane, and coronal plane as locally enlarged depicted in Fig. 8). Therefore, four metrics (DC, IoU, HD, and PA) were used for quantified evaluation of the segmentation results, and their results among five testing cases are given in Table 6. The mean DC of all cases is 0.953 ± 0.014, and the mean IoU is found to be 0.911 ± 0.025. HD represents the distance between each surface voxel of the segmented surface from the closest surface voxel in the ground-truth, the larger the performance is worse. Case 15 has the largest HD, which is 5.443 ± 4.509 mm. HD in case 14 is the smallest and can reach 3.156 ± 1.241mm. The mean PA result of all testing cases is impressive, which can reach up to 0.998 ± 0.001. Since PA considers the TN i.e., true negative pixels or voxels which represent background in the ROI and occupy most of the space in the ROI, the large value of PA most likely credited to these pixels or voxels were correctly predicted.

**Table 6:** Segmentation results of different cases.

Metrics	DC	IoU	HD (mm)	PA
Case11	0.951 ± 0.017	0.908 ± 0.031	3.177 ± 1.156	0.998 ± 0.001
Case12	0.955 ± 0.011	0.914 ± 0.019	4.063 ± 1.099	0.997 ± 0.001
Case13	**0.950 ± 0.013**	**0.906 ± 0.023**	4.227 ± 2.637	0.998 ± 0.001
Case14	**0.958 ± 0.010**	**0.919 ± 0.019**	**3.156 ± 1.241**	0.998 ± 0.001
Case15	0.952 ± 0.018	0.909 ± 0.032	**5.443 ± 4.509**	0.997 ± 0.001
All	0.953 ± 0.014	0.911 ± 0.025	4.013 ± 2.128	0.998 ± 0.001

Bold values indicates maximum and minimum values of the corresponding column or row.

**Figure 9:**
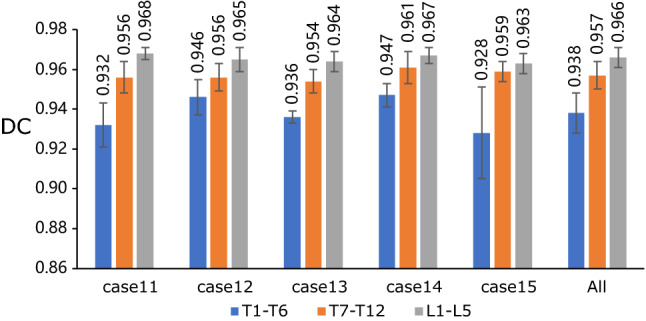
Evaluation on three groups: upper thoracic, lower thoracic and lumbar spine.

Additionally, the vertebrae were grouped into three groups according to their anatomy property: (1) the upper thoracic group: from T1 to T6, (2) the lower thoracic group: from T7 to T12, and (3) the lumbar spine group: from L1 to L5. The results of DC regarding these three groups are shown in Fig. 9. The best result appears in the lumbar spine group that belongs to case 11, and the corresponding DC is 0.968. In contrast, the upper thoracic group of case 15 has the worst result of DC, which is 0.928. For all testing cases, DC on the lumbar spine has a better result, followed by lower thoracic, upper thoracic. It may be primarily influenced by two factors: (1) the vertebra size at the upper thoracic level is smaller than that at the lumbar level, and the bone density is lower as well. (2) The interfaces with surrounding structures are more complex at the upper thoracic level, particularly at the costovertebral junctions that connect the ribs and the vertebrae^25^. The comparison results between our method and some traditional methods on these three groups are presented in Table 7. The overall mean result of 0.953 ± 0.014 in term of DC is better than other methods. On the three groups, our results of 0.938 ± 0.010, 0.957 ± 0.004, 0.966 ± 0.005 also all exceeds the respective result presented by Hammernik et al^40^ and Korez et al^41^.

**Table 7:** Comparison with some state-of-the-art traditional methods.

DC	Hammernik et al.^40^	Korez et al.^41^	Our method
Upper thoracic	0.89 ± 0.05	0.913 ± 0.010	0.938 ± 0.010
Lower thoracic	0.95 ± 0.02	0.936 ± 0.005	0.957 ± 0.007
Lumbar spine	0.96 ± 0.02	0.944 ± 0.020	0.966 ± 0.005
Mean	0.93 ± 0.04	0.931 ± 0.020	0.953 ± 0.014

Several state-of-the-art deep-learning algorithms for vertebrae segmentation using the same thoracolumbar spine CT dataset were also compared with our results as listed in Table 8. Since Janssens et al.^23^ only segmented the ROI of lumbar, the segmentation results of lumbar is listed as row “Lumbar” for comparing separately and it shows that our segmentation result of DC in lumbar spine exceeds the method presented by Janssens et al.^23^. In addition, our segmentation method exceeds the method presented by and Lessmann et al. (2018)^42^, but slightly worse than the performance of Lessmann et al. (2019)^24^. As mentioned in Lessmann et al. (2019)^24^, they trained their network on an Nvidia Titan X GPU taking about 4-5 days for 100,000 iterations. Compared with that, it only took 10 hours to train our network on Nvidia GTX 1080Ti with 30 epochs for vertebrae localization and 50 epochs for vertebrae segmentation respectively. Therefore, our method requires lower GPU equipment and training time. Besides, our accuracy does not decrease significantly.

**Table 8:** Comparison with several deep-learning state-of-the-art methods.

DC	Janssens et al.^23^	Lessmann et al.^42^	Lessmann et al.^24^	Ours
Lumbar	0.957 ± 0.08	–	–	0.966 ± 0.005
Mean	–	0.948 ± 0.016	0.963 ± 0.013	0.953 ± 0.014

To further evaluate the generalizability and the performance on pathological cases, we conducted experiments on xVertSeg dataset in terms of evaluation on LE, DR and DC. The experimental results are listed in the former three data columns of Table 9 that, the mean LE is 4.12 ± 2.31, the DR is 100 % i.e., all vertebrae are identified in the cropped ROI, the mean DC is 0.877 ± 0.035. We also compared our results of DC with Chuang et al.^43^ and Lessman et al.^42^ on xVertSeg dataset as shown in the last three columns of Table 9. It shows that the DC of L2, L3 are better than other methods and the mean DC exceeds Lessman et al.^42^ but slightly worse than Chuang et al.^43^. Compared with the mean DC on CSI dataset, the mean DC on xVertSeg dataset is a little worse. It may be primarily influenced by two factors: (1) The xVertSeg dataset is a lumbar dataset and the amount of the vertebare for the network to learn is much less than CSI dataset. (2) The xVertSeg dataset contains vertebare with fractures of different grade. Accordingly, the experimental results on xVertseg dataset could also be analyzed from the perspective about non-fractured vertebrae and vertebrae with fractures of different grade to evaluate the performance on pathological cases. The results of DC are separately listed according to the fractured grade in Table 10. Column “Grade” shows the different grade of vertebra and the higher the grade, the more severe fractured of the vertebra. The Column “Amount” shows the amount of vertebra with different grade used for evaluation. In Table 10, grade 3 has the minimun DC, grade 0 and grade 2 have the similar higher DC. Generally, evaluation on xVertSeg dataset validates the generalizability and the performance on pathological cases of the proposed method.

**Table 9:** Evaluation on xVertSeg dataset in terms of location error (LE), detection rate (DR) and dice coefficient (DC).

	LE	DR	DC		
	Ours	Ours (%)	Ours	Chuang et al.^43^	Lessman et al.^42^
L1	**4.33 ± 2.85**	100%	0.873 ± 0.058	**0.883**	0.881
L2	3.60 ± 2.11	100%	**0.877 ± 0.042**	0.861	0.874
L3	3.92 ± 1.66	100%	**0.896 ± 0.020**	0.862	0.816
L4	**3.96 ± 1.75**	100%	0.877 ± 0.029	**0.892**	0.724
L5	4.76 ± 3.56	100%	0.860 ± 0.012	**0.926**	0.881
Mean	4.12 ± 2.31	100%	0.877 ± 0.035	0.885	0.835

Bold values indicates maximum and minimum values of the corresponding column or row.

**Table 10:** DC of non-fractured vertebrae and vertebrae with fractures of different grade.

Grade	Amount	Mean DC
0	10	0.884 ± 0.019
1	8	0.869 ± 0.056
2	3	**0.886 ± 0.021**
3	4	**0.867 ± 0.026**

Bold values indicates maximum and minimum values of the corresponding column or row.

## Conclusion

In this paper, a two-stage Dense-U-Net approach was developed for vertebrae localization and segmentation. For vertebrae localization, first, we proposed a novel method to refine the original dataset by creating sparse annotation of centroids and converting them to dense labels. Then, 2D-Dense-U-Net was performed to train and test with 2k+1 CT transversal slices and their corresponding dense labels. Finally, an aggregating method was adopted to estimate each final vertebra centroid from the predicted dense result. The experimental results on CSI dataset demonstrated the mean location error of the predicted vertebra centroid is 1.69 ± 0.78 and the detection rates of all testing cases are 100% in identified ROIs, which showed that all these ROIs could be used for subsequent segmentation task. For vertebrae segmentation, data augmentation methods incl. elastically deform and Gaussian noise were applied on the identified ROIs. Then, the 3D-Dense-U-Net was trained and tested with these ROIs as the input. The experimental results on CSI dataset in terms of DC, IoU, HD, and PA demonstrated that we successfully and efficiently finished instance segmentation of the vertebrae. Particularly, our method shows great performance of 0.953 ± 0.014 in DC and the results of DC exceed some traditional state-of-the-art methods on the three groups of the spine. Moreover, we compared our method with some deep-learning state-of-the-art methods for vertebrae segmentation, it showed that we also exceeded methods presented by Janssens et al.^23^ and Lessmann et al. (2018)^42^ but slightly worse than Lessmann (2019)^24^. Furthermore, evaluation on xVertSeg dataset validates the generalizability and the performance on pathological cases.

The proposed method was based on the Dense-U-Net that combined with dense blocks and long skip connections that are advantageous to improve the accuracy of localization and segmentation^21^. However, there are still some directions that could be improved in the future. First, 3D-Dense-U-Net used at the second stage could be optimized by combining the attention model. Second, since the mean DC of the upper thoracic showed a little worse than that of the lumbar spine, further investigation is necessary to improve the segmentation of the upper spinal column. Finally, hardware generations with larger memory will enable training of larger networks and higher resolution which might lead to further performance improvements.

## Data Availability

The datasets built during the current study are available from the corresponding author on reasonable request.
